# Impacts of host gender on *Schistosoma mansoni* risk in rural Uganda—A mixed-methods approach

**DOI:** 10.1371/journal.pntd.0008266

**Published:** 2020-05-13

**Authors:** Suzan C. M. Trienekens, Christina L. Faust, Keila Meginnis, Lucy Pickering, Olivia Ericsson, Andrina Nankasi, Arinaitwe Moses, Edridah M. Tukahebwa, Poppy H. L. Lamberton

**Affiliations:** 1 Institute of Biodiversity, Animal Health and Comparative Medicine, College of Medical, Veterinary and Life Sciences, University of Glasgow, Glasgow, United Kingdom; 2 Wellcome Centre for Integrative Parasitology, College of Medical, Veterinary and Life Sciences, University of Glasgow, Glasgow, United Kingdom; 3 Institute of Health & Wellbeing, College of Social Sciences, University of Glasgow, Glasgow, United Kingdom; 4 Economics Division, University of Stirling Management School, Stirling, United Kingdom; 5 Vector Control Division, Ministry of Health, Kampala, Uganda; World Health Organization, INDONESIA

## Abstract

**Background:**

The World Health Organization identified Uganda as one of the 10 highly endemic countries for schistosomiasis. Annual mass drug administration (MDA) with praziquantel has led to a decline in intensity of *Schistosoma mansoni* infections in several areas. However, as hotspots with high (re)infection rates remain, additional research on risk factors and implementing interventions to complement MDA are required to further reduce disease burden in these settings. Through a mixed-methods study we aimed to gain deeper understanding of how gender may impact risk and reinfection in order to inform disease control programmes and ascertain if gender-specific interventions may be beneficial.

**Methodology/Principal findings:**

In Bugoto, Mayuge District, Eastern Uganda we conducted ethnographic observations (n = 16) and examined epidemiology (n = 55) and parasite population genetics (n = 16) in school-aged children (SAC), alongside a community-wide household survey (n = 130). Water contact was frequent at home, school and in the community and was of domestic, personal care, recreational, religious or commercial nature. Qualitative analysis of type of activity, duration, frequency, level of submersion and water contact sites in children showed only few behavioural differences in water contact between genders. However, survey data revealed that adult women carried out the vast majority of household tasks involving water contact. Reinfection rates (96% overall) and genetic diversity were high in boys (pre-He = 0.66; post-He = 0.67) and girls (pre-He = 0.65; post-He = 0.67), but no differences in reinfection rates (p = 0.62) or genetic diversity by gender before (p = 0.54) or after (p = 0.97) treatment were found.

**Conclusions/Significance:**

This mixed methods approach showed complementary findings. Frequent water exposure with few differences between boys and girls was mirrored by high reinfection rates and genetic diversity in both genders. Disease control programmes should consider the high reinfection rates among SAC in remaining hotspots of schistosomiasis and the various purposes and settings in which children and adults are exposed to water.

## Author summary

Globally, over 230 million people are infected with schistosomiasis, an infectious disease caused by parasitic helminths. Humans can get infected when they contact water which contains *Schistosoma* parasites. Although the disease can be treated with a drug, people get rapidly reinfected in certain high-transmission settings. Drug treatment alone may not be sufficient to eliminate this disease and additional interventions such as health promotion or improvements in water and sanitation need to be scaled up. To provide recommendations to these control programmes we carried out interdisciplinary research in Eastern Uganda to understand the influence of gender on schistosomiasis risk. We found that the water contact behaviour of boys and girls is quite similar, and we did not see differences in reinfection or genetic diversity of the parasite between boys and girls. Differences in water contact between genders is greater in adults, and further research is required for these individuals. In this setting, infection rates are high in school-aged children and there are no differences between genders. These results emphasise improved control efforts for all school-aged children in communities like these. Our interdisciplinary approach provided complementary findings. Such an integrated approach can therefore have more power to meaningfully inform policy on schistosomiasis control.

## Introduction

Globally more than 230 million people are infected with schistosomiasis [1] causing an estimated 1.9 million Disability-Adjusted Life Years (DALYs) [2]. Schistosome parasites are acquired when humans contact freshwater that contains infective cercariae which actively penetrate the skin. These cercariae develop into adult worms, pair off, and sexually reproduce. Eggs are then excreted in human faeces or urine. When they reach freshwater they hatch into miracidia which penetrate intermediate snail hosts where they asexually reproduce. Snails then release thousands of free-swimming cercariae back into the water. The World Health Organization (WHO) recommends mass drug administration (MDA) with praziquantel in endemic areas to prevent morbidity and reduce transmission [3]. There is now a push to supplement MDA with additional interventions, such as water, sanitation and hygiene (WASH) and health promotion campaigns, to improve outcomes for control in highly endemic areas, and to help move towards eradication in areas where MDA has been successful [4]. Understanding how host gender may influence the risk of schistosomiasis infections and reinfections could enable more targeted integrated control efforts. This in turn could increase effectiveness while minimising resources required.

*Schistosoma* infection occurs only through contact with infectious freshwater. Therefore, understanding the different aspects of water contact is essential for understanding the risk of infection and subsequent disease. Water contact can occur through a range of host activities such as during household tasks, activities related to hygiene, occupational activities (e.g. commercial clothes washing, fishing) and leisure pursuits. It is well established that there are differences in water contact behaviours between men and women, and this can be influenced by cultural, professional and religious factors [5]. For example, Pearson [6] observed that men and women in a fishing village in north western Uganda contact water for different tasks, at different times of the day and for different durations. Because cercariae emergence most frequently occurs at noon [7, 8], understanding the times of day that people contact water is important for evaluating risk in addition to duration and frequency. A study in Kenya however showed that participants in focus groups said no gender was at higher risk, as modernisation means that women and men now carry out more similar tasks in their community [9]. Limitations of behavioural studies are that they are often a proxy for disease risk and they do not directly measure infectious risk of cercariae, therefore understanding human behaviours may not be sufficient for understanding infections and subsequent disease. Complementing data on water contact behaviour with parasitological data could contribute to understanding possible outcomes of water contact, particularly in respects to gender-specific practices.

Gender has been shown, in some settings, to impact likelihood of infection, rates of reinfection, and the genetic diversity of schistosomes that individual humans harbour. Praziquantel treatment clears between 80–95% of *Schistosoma mansoni* (main cause of intestinal schistosomiasis) adult worms in an individual [10] but treatment does not prevent reinfection. Reinfection is a particular challenge in areas with high rates of transmission, where parasite reservoirs in snail vectors and untreated humans can quickly reinfect treated individuals [11]. A meta-analysis of 32 studies on *Schistosoma* spp. reinfection found that males (children and adults) were more often reinfected between 6 and 12 months after treatment with praziquantel than females (pooled OR: 1.45, p = 0.04 [12]). However, two recent studies looking at reinfection with *S*. *mansoni* in school-aged children specifically (ages 6–14 years), in endemic settings in Tanzania and Brazil, found no difference in reinfection between boys and girls at 5, 8 [13], and 12 months [11] after treatment.

Another way in which to assess infection risk is to measure the genetic diversity of parasites within hosts [14]. Higher genetic diversity suggests exposure to more, genetically different parasites—this can occur through multiple exposures at the same location or exposures across geographically different areas. This genetic diversity can be confounded by a host’s immune response [15], but it gives a window into an individual host’s long-term exposure as well as their subsequent contribution to further transmission. Some studies that investigated genetic diversity of *S*. *mansoni* parasites by gender have found no differences by host gender across whole populations [16, 17] or school-aged children [18]. However, a study that examined a greater number of SAC than previous studies found small, but significant, differences in genetic diversity of parasites when considering age and gender of SAC in Mayuge District, Uganda [19]. This suggests that people of different gender can be exposed to different parasites and that differences in immunity may alter within-host diversity as children age. However, these studies have been few and warrant further investigation given the differences in epidemiology, potential risk behaviours, and results from studies measuring genetic diversity of *Schistosoma haematobium* finding higher genetic diversity in male SAC. Using these genetic methods alongside parasitological, epidemiological and anthropological methods may help to elucidate the complex aspects associated with *S*. *mansoni* risk and infection.

As previous research on the gender-specific aspects of schistosomiasis is limited [20], we aimed to gain a deeper understanding of gender differences in exposure risk behaviours and how these manifests in schistosome epidemiology using a mixed methods approach in Mayuge District, Eastern Uganda. WHO has identified Uganda as one of the ten most highly endemic countries for schistosomiasis [4]. In 2003, the national control programme began to roll out annual MDA in schools and moved to community-based MDA in 2007 in high endemic districts. While MDA has been effective in some regions [21, 22], despite over a decade of MDA, *S*. *mansoni* prevalence remains high in Mayuge District at >80% for both genders. Many school-aged children (SAC) are rapidly reinfected post-treatment [23] and mean infection intensities remain high in this group [24].

We use ethnographic observations, a household survey, epidemiological survey and parasite population genetics in one community comprised of two villages in Mayuge District, to assess differences by gender in water contact, reinfection and parasite genetic diversity.

## Methods

### Study site

All field research was conducted in Bugoto, Mayuge District, Uganda, which has approximately 3,500 inhabitants, divided over two villages. Bugoto A is a densely populated village situated on the shores of Lake Victoria and the majority of the adult population are fisherfolk or fishmongers [25]. Bugoto B, to the north and inland of Bugoto A, is less densely populated, and most inhabitants are farmers. There is a health clinic in Bugoto A, serving both villages. The main language spoken is Lusoga, a language spoken across the Busoga region in the south east of Uganda.

### Terminology

We will use the term ‘gender’ throughout the discussion of our methods and results to describe self-reported identity of study participants and acknowledge the sociocultural aspects of exposure and infection, and limit discussion of our results to gender differences. Differences based on biological ‘sex’ are explicitly discussed in the introduction and discussion and reflect the data presented in those studies.

### Ethnographic observations

To compare water contact behaviour between different risk groups, participants for the ethnographic observations were selected from a larger cohort (n = 274), recruited as part of the ongoing SCHISTO_PERSIST project, comprising SAC with an equal age/gender distribution. This cohort was recruited in March 2017 at Bugoto Lake View Primary School, the largest primary school in the community situated between Bugoto A and Bugoto B. Samples were taken from this cohort at four time points in 2017; March, September, October and December, with treatment administered after the September sample collection. Detected infection with schistosome parasites and mean infection intensities were recorded at every time point. In March 2018, eight children with no/very low and eight children with high baseline and reinfection intensities were recruited from the larger cohort for ethnographic observation. An effort was made to equally represent ages, gender and village of residence across each group using unique identifiers.

Ethnographic data were collected from the 5^th^ to 24^th^ March 2018, covering school days, and the 19^th^ September to 10^th^ October 2018, covering non-school days. A local village health team member was recruited to carry out translations between the researcher and the community. Observations of individual children were conducted from 10.30am (beginning of first school break, for the first data collection period) or 9.00am (for the second data collection period) to approximately 9.00pm. The researcher and translator spent the day accompanying and observing the child in their daily activities (including all water contact moments) and participating where possible and appropriate. Participation excluded high-risk activities such as water contact, and participation and observation excluded privacy-sensitive (toilet/shower use), culturally inappropriate (mosque attendance) or disruptive (class attendance) activities. In addition, we asked the child if they had any water contact on the day before our arrival. Data collection stopped when the child went to bed or no further water contact activities would be carried out before going to bed. Notes were taken during the day and later written up in detail. Transcripts were reviewed, coded and categorised using NVivo software [26], using thematic analysis [27], with a coding frame based on previously published literature complemented with codes emerging during the coding process.

### Household survey

A household survey was administered in Bugoto A and B in February 2019 as part of a wider study on attitudes and behaviours for different water, sanitation and hygiene (WASH) interventions aimed at reducing *S*. *mansoni* transmission. The sampling methods have been described elsewhere [28]. The required sample size was 125 and based on the latest national census of the village of 2018 the household sampling interval was therefore six. We interviewed one adult (≥18 years old) respondent per household and aimed to have an equal gender distribution among respondents. One of five research assistants read out the survey to each participant in the local language and responses were collected on tablets using Sawtooth Computer Assisted Personal Interview software. Collected data for this study included demographic and socio-economic characteristics, knowledge of schistosomiasis, and water collecting practices. Data were cleaned with STATA software [29], and frequency and percentages were calculated. Gender differences were calculated with exact binomial tests and two-proportions z-tests.

### Epidemiology diagnostics cohort

A random sample of 55 *Schistosoma mansoni* positive children (gender matched and evenly distributed across the 6–14 years age range) were selected from the SCHISTO_PERSIST cohort. The individuals were all positive for *S*. *mansoni* in October 2017 and were treated with 40 mg praziquantel per kilogram body weight. These individuals were resampled up to twice per week for five months following treatment (S1 Fig). At each timepoint, children were recruited at 9:00am and asked to give a single stool sample for duplicate thick smear Kato-Katz (KK) [30]. If an individual was diagnosed as having over 100 eggs per gram (epg) at nine weeks post-treatment, she/he was retreated with 40 mg/kg praziquantel (n = 3) for ethical reasons. At pre-treatment and five months post-treatment, children were sampled for three consecutive days to improve diagnostic accuracy [31]. Significant predictors of infection intensity (measured as epg per slide) were evaluated using a generalised linear mixed effects model with a negative binomial distribution to account for overdispersion of these data [32]. Gender and relative treatment (pre-treatment vs five months post-treatment) were used as fixed effects and unique child ID included as a random effect to account for repeated samples from individuals from multiple slides per day and days per time point. Models were created using the package *lme4* [33] in R v. 3.5 [34]. Egg counts (as measured by KK) are highly variable between days, particularly in individuals with low infections [35, 36], so clearance of schistosomiasis was defined as two consecutive days of 0 epg in duplicate KK slides. The time to reinfection was identified as the first day that eggs were detected in stool following this clearance event. We used survival analysis methods to evaluate the time to event (here, reinfection rather than death) to determine if there are significant differences between genders in the proportion reinfected or the time to reinfection. The Kaplan-Meier method was used to fit the time to reinfection and estimate 95% confidence intervals (CI), and significant differences were evaluated using a log-rank test. The survival analysis was conducted with the *survivfit* function within the package *survival* [37] in R v. 3.5 [34].

### Genetic diversity of schistosomes

To evaluate the genetic diversity of *S*. *mansoni* within individual children, miracidia were collected throughout the epidemiological survey. Collection protocols followed established methods [38], but briefly, after stool samples were submitted for KK, the remainder of the sample was washed through a Pitchford funnel with spring water [39]. The washed sample, potentially containing eggs, was left overnight in the dark and then exposed to indirect sunlight the following day. Individual miracidia were picked up in 2.0–2.5 μl of water with a micropipette under a stereomicroscope and placed on Indicating FTA Whatman cards. Samples were left to dry a minimum of one hour before being stored and transported in sealed plastic bags with desiccants. Data presented here are from miracidia from eight boys and eight girls collected in October 2017 (pre-treatment) and March 2018 (five months post-treatment). These individuals did not overlap with children selected for the ethnographic observations; rather these individuals were chosen because we were able to obtain miracidia from children at both timepoints (details in S2 Table).

DNA from individual miracidia was eluted from FTA cards using established protocols [40]. Seventeen microsatellite loci were amplified per miracidium using two separate PCR reactions (S1 Table). Type-IT microsatellite PCR kits (Qiagen) were used according to the manufacturer’s protocol for each multiplex PCR. Each PCR reaction was carried out in 12.5μl total volume using 2μl of eluted DNA from each miracidia. Multiplex amplicons were visualised on a 2% agarose gel stained with Sybr Safe (Invitrogen). Successfully amplified DNA from miracidia was diluted 1:50 in LIZ500 Size Standard (ThermoFisher) and HiDi Formamide before sequencing on an Applied Biosciences. Allele peaks were visualised and called in GeneMapper (ThermoFisher).

Genetic diversity measures were calculated for all miracidia and loci for each individual at each time point. Observed heterozygosity (Ho), expected heterozygosity (He), and allelic richness were calculated in *poppr* v. 2.8.1 [41]. Genetic diversity pre- and post-treatment was compared used a Wilcoxon signed rank test implemented with the function *wilcox*.*test* in R. The number of populations and the ordination of different parasite genotypes was investigated using Discriminant Analysis of Principal Components (DAPC) within the *adegenet* package [42, 43]. Variation among genders and timepoints was summarised by calculating Nei’s G_ST_ with functions in the package *mmod* [44].

### Ethical clearance

Ethical approval for the survey and ethnographic research was granted by the Ugandan National Council for Science and Technology Social Sciences (reference: UNCST-SS 4241) and the University of Glasgow College of Social Sciences (reference: 400160134). Methods for the epidemiological and genetic data were reviewed and approved by the Vector Control Division Research Ethics Committee (VCDREC/062), the Uganda National Council of Science and Technology (UNCST-HS 2193) and the University of Glasgow Medical, Veterinary and Life Sciences Research Ethics Committee (200160068). Community consent was obtained from the District Education Officer, the District Health Officer and the Chairmen of the study villages. Individual written consent (signature or thumb print) was obtained from all participants 18 and over. Participants under 18 years old provided assent and additional consent was provided from a parent or guardian. Praziquantel treatment was not conditional on participation in the study or giving consent and all individuals could leave the study at any time without affecting their drug treatment.

## Results

### Few differences in water contact between genders observed during ethnographic observations

Nine females and seven males, aged between 6 and 14 years old (median 10 years) were selected for ethnographic observations. Nine children were living in Bugoto A, seven children in Bugoto B. Seven identified as Muslim and nine as Christian (different branches). All consented to participate in the study. Two children were lost to follow up for the second data collection period; these were replaced by children with similar demographic characteristics. Differences in water contact by gender were assessed by types of activity, frequency, contact sites, level of submersion and duration of contact. No major cultural events that may have influenced behaviours were observed to have taken place during the data collection period.

We observed a range of activities carried out by the participating children that involved contact with lake water, in various settings and for different purposes. The main exposures to water were household tasks including fetching water, washing plates and jerrycans, washing clothes and flip flops, washing food items (potatoes, fish, vegetables, fruit), mopping by hand and personal care such as bathing (self and helping younger or disabled household members) and washing hands. Other types of water exposure were of a recreational (swimming) or a religious nature (washing hands, feet and face before attending the mosque). Occasional water contact also occurred at school, as children took turns in fetching water for the cook to prepare meals for students and when they washed their cup/plate after eating. Children were not seen to contact water in occupational settings such as working in rice paddies or helping with fishing, however some children carried out ‘commercial fetching’, fetching water for others in the community, receiving a small amount of money in return. Lake water was not observed to be processed by filtering, purification or sun exposure although large amounts of soap are used for washing plates, clothes and bathing. All water for drinking was fetched from a borehole or (paid-for) tap. Rain was considered ‘clean’ water and safe for drinking and household use by participants and their families, however it was not regularly collected due to the absence of water containers or tanks.

Children fetched lake water for the household between one and three times per day, and we found that girls fetched water for the household more often (on 11 of 14 observation days) than boys (on 10 of 18 days). Girls also more often fetched water more than once per day (on seven observation days vs four in boys); most of these children who collected water multiple times a day lived in Bugoto A, which is located closer to the lake. Commercial fetching was only carried out by children in Bugoto A, probably due to the higher number of businesses requiring water and the short distance to the lake. Two boys and two girls were found to carry out commercial fetching, up to five times per day.

Most children washed plates during the observation days, either one or two times per day. Although many children and parents said that children take turns in washing plates, more girls (on 12 out of 14 days) were observed to carry out this task than boys (on 10 out of 18 days). A few children washed clothes, however while spending time at participants’ homes, adult women seemed to be mainly responsible for this household chore. Washing of food items and mopping of the house was carried out by two girls and one boy. Most children bathed once a day, those who did not mentioned that the temperature in the evening was too low to bathe outside. Four girls bathed twice a day, however reasons for this are unknown. Hand washing was performed regularly by all children, mainly after meals, likely due to the fact that the hands are used for eating. The highest frequencies of hand washing were seen with children who were not in school, who either carry out many household tasks or handle different items at jobs.

Four of the boys engaged in swimming (including playing in the water, diving off boats, submerging to cool down during a hot day or small races) during the observations whilst none of the girls did. In addition, no other girls of the community were seen swimming at these sites. The translator confirmed that only a few girls swim and all children stop swimming when they reach puberty. Swimming activities ranged in frequency from one to four times per day.

Only one boy was observed to wash his hands, feet and face before every visit to the mosque. The other male Muslim participants did not visit the mosque at all during the observations and during the two data collection periods no women were seen entering the mosque or praying elsewhere.

No patterns of difference between gender were found when comparing sites of fetching water. Participants mentioned that their site of choice was closest to their home or cleanest and it was observed they consistently accessed water at the same site. Only when their preferred source dried up in the dry season, or water was considered too dirty by the child, would they move to another site. Observations while accompanying children to water contact sites did not reveal any access restrictions by gender and both genders were found to contact water at the sites. Washing plates, clothes, food items, body or hands were all carried out at the house compound with water previously collected at the lake.

The level of submerging and duration of water contact was not noticeably different between boys and girls for the activities both genders carried out, as all children showed similar approaches to the various activities. Level of submerging was more related to activity (fetching, swimming) and varying levels of submerging (until ankles, knees or hips pulling trousers or skirt up) during fetching were only seen when a participant sought for clearer water which was often further from the shore. Duration of fetching water, cleaning food items and washing plates and clothes was mainly based on the number of items. Children of both genders who carry out these activities tended to have similar and a continuous pace until the work was complete, without visible rushing or loitering. The weather affected the duration of some activities, for example bathing was observed to be rushed when the temperature was low, whilst on days with high temperatures boys were seen swimming for extended lengths of time.

### Adult females responsible for the majority of household tasks involving water contact

A total of 130 household heads (52% female) participated in the household survey: 85 (65%) in Bugoto A and 45 (35%) in Bugoto B, answering on behalf of their whole household. During the ethnographic observations we identified fetching water and washing clothes as two key household tasks involving water exposure. Respondents were therefore asked who fetches the majority of water and who washes the majority of clothes for the household, specified by gender and age. In 106 (82%) households one person fetches the majority of the water, in 24 (18%) households this was between two to four persons. Almost all respondents (n = 127, 98%) reported that a member of the household, rather than someone outside the household, fetches the majority of the water. The remainder said other members of the community, unrelated to household members and over 18 years old, fetch water for their household. Of these, two were paid for their help.

For 118 (91%) households, there was one main person responsible for washing of clothes, for 12 households this was more than one person. Washing of clothes is mostly undertaken by household members, with only one respondent reporting clothes washing by other members of the community. No respondents reported that neighbours, or other family members not living in the household, fetched water or washed clothes for them.

For both fetching water and washing clothes, women were mainly responsible (68% and 89% of households respectively; Table 1), and especially those over 18 years old. Men were less often reported responsible for fetching (15%) and washing clothes (4%), whilst men and women shared the responsibility for the tasks in 17% and 7% of households respectively. In households with a single gender responsible for these tasks, women significantly more often fetched water (probability of 0.82, CI [0.74–0.89], p<0.001) and washed clothes (probability of 0.96, CI [0.91–0.99], p<0.001) as compared to an equal distribution between the two genders. No differences in the gender distribution for these two household tasks were found by demographic or socio-economic characteristic (Table 2).

**Table 1:** Key household activities involving water contact by gender and age, as reported by household head (n = 130).

Activity	Female only				Male only				Mixed gender				Total
	<18y n(%)	≥18y n(%)	Mixed ages n(%)	Female total n(%)	<18y n(%)	≥18y n(%)	Mixed ages n(%)	Male total n(%)	<18y n(%)	≥18y n(%)	Mixed ages n(%)	Mixed gender total n(%)	
**Fetching water**	17 (13.0)	71 (54.6)	1 (0.8)	**89 (68.4)**	4 (3.1)	14 (10.7)	1 (0.8)	**19 (14.6)**	7 (5.4)	0	15 (11.6)	**22 (16.9)**	**130 (100)**
**Washing clothes**	10 (7.7)	103 (79.2)	3 (2.3)	**116 (89.2)**	0	5 (3.8)	0	**5 (3.8)**	1 (0.8)	0	8 (6.2)	**9 (6.9)**	**130 (100)**

**Table 2:** Gender distribution for fetching water and washing clothes by demographic and socio-economic characteristics (n = 130).

Demographic / socio-economic characteristic		Fetching water			Washing clothes		
	Total	Female n(%)	Male n(%)	Mixed gender n(%)	Female n(%)	Male n(%)	Mixed gender n(%)
**Village** Bugoto A Bugoto B	**85** **45**	60 (71) 29 (64)	10 (12) 9 (20)	15 (18) 7 (16)	76 (89) 40 (89)	3 (4) 2 (4)	6 (7) 3 (7)
**Household size** 1–6 members >6 members	**66** **64**	51 (77) 38 (59)	10 (15) 9 (14)	5 (8) 17(27)	58 (88) 58 (91)	5 (8) 0	3 (5) 6 (9)
**Religion** Christian Muslim	**64** **66**	49 (77) 40 (61)	6 (9) 13 (20)	9 (14) 13 (20)	58 (91) 58 (88)	3 (5) 2 (3)	3 (5) 6 (9)
**Education** No education Primary education Secondary or higher education	**28** **69** **33**	23 (82) 45 (65) 21 (64)	3 (11) 10 (14) 6 (18)	2 (7) 14 (20) 6 (18)	26 (93) 60 (87) 30 (91)	1 (4) 2 (3) 2 (6)	1 (4) 7 (10) 1 (3)
**Income (UGX/day)** <3,000 ≥3,000	**57** **73**	42 (74) 47 (64)	7 (12) 12 (16)	8 (14) 14 (19)	53 (93) 63 (86)	1 (2) 4 (5)	3 (5) 6 (8)
**Heard of schistosomiasis** Yes No I don’t know	**130** **0** **0**	89 (100) 0 0	19 (100) 0 0	22 (100) 0 0	116 (100) 0 0	5 (100) 0 0	9 (100) 0 0
**Identifies water contact as exposure risk** Yes No	**76** **54**	53 (70) 36 (67)	11 (14) 8 (15)	12 (16) 10 (19)	69 (91) 47 (87)	2 (3) 3 (6)	5 (7) 4 (7)

The occupation involving most water contact was fishing. Twenty-nine per cent of the population in Bugoto A reported fishing as their occupation, and 9% of those living in Bugoto B. There was a significant difference between genders; 28 of the 62 men (45%) were fisherfolk compared to one of 68 women (χ^2^ = 33.23, CI [0.29–0.58], p<0.001). Water contact can also occur when farming, reported by 35% of those in Bugoto A compared to 64% in Bugoto B, however a similar proportion of men and women reported this as their occupation (45% of men and 46% of women (χ^2^<0.001, CI [-0.17–0.18], p = 1).

### Rapid reinfection with *S. mansoni* in both genders

Fifty-four SAC (out of 55) gave samples for at least 40% of sample collection days throughout the five months and were included in the analysis. Both genders had a moderate mean infection intensity pre-treatment: 162.3 epg in males and 122.5 epg in females. At five months post-treatment, the mean infection intensity pre-treatment was lower in each gender; 99.8 epg in males and 80.8 epg in females. Our mixed-effects modelling results suggest that drug treatment affects observed infection intensity, with infection intensity significantly higher pre-treatment than post (slope estimate = 1.03, z-value = 3.88, p = 0.0001). However, gender was not a significant predictor of infection intensity (p = 0.37).

**Fig 1:**
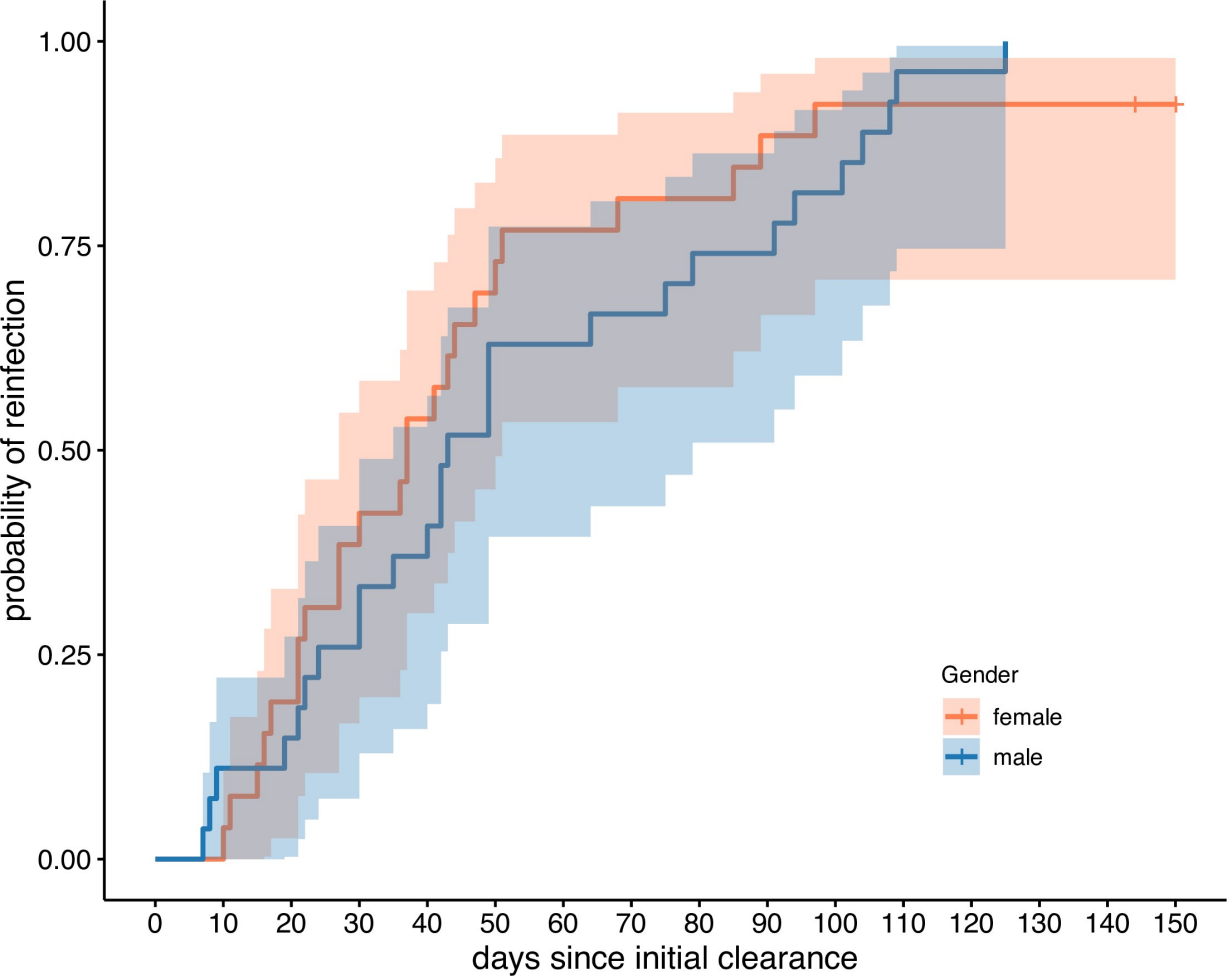
Reinfection by gender. Reinfection curves and 95% confidence intervals by gender for the cohort of 54 children that were sufficiently sampled during the longitudinal study. There is no significant difference in reinfection rates or time to reinfection between genders.

Fifty-two out of the 54 (96%) individuals were identified as reinfected during the five month follow-up period (egg positive after a period of clearance). Among these reinfected individuals, the period of no egg production varied from seven to 125 days. All males were reinfected in the time period observed and the mean time to reinfection was 54.4 days. All but two females were reinfected within the observation period and the mean time to reinfection for females was 39.3 days. Despite this difference, Kaplan-Meier curves found no significant difference in the likelihood of reinfection between genders (Fig 1; p = 0.62).

### No differences in within-host parasite genetic diversity between genders

**Fig 2:**
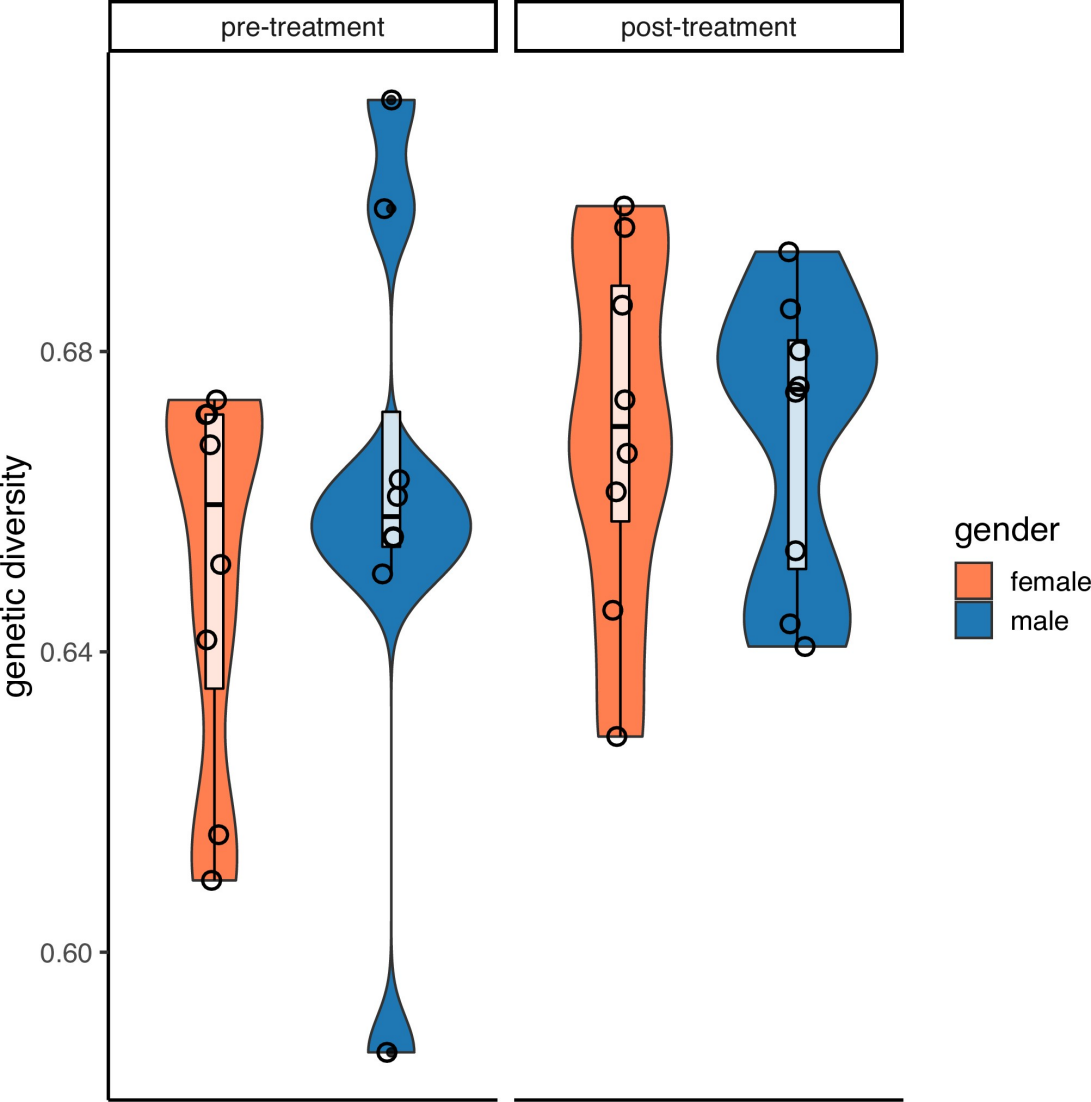
Mean genetic diversity by gender before and five months after treatment. There are no significant differences in average genetic diversity (measured as expected heterozygosity) between male and females, either before or after treatment. Violin plots show distribution of genetic diversity and internal boxplots show mean and first and third quartile range per gender and timepoint. Mean genetic diversity per child is shown by individual open circles.

A total of 386 miracidia were successfully genotyped from the 16 children (eight males, eight females) at pre-treatment and five months post-treatment. The average genetic diversity of *S*. *mansoni* collected from female hosts (pre-He = 0.65; post-He = 0.67) was not significantly different to those collected from male hosts (pre-He = 0.66; post-He = 0.67) before (Fig 2; t = -0.62; p = 0.54) or after treatment (t = 0.12; p = 0.97). Parasite genetic diversity pre-treatment was not significantly correlated with parasite genetic diversity post-treatment in the same host (p = 0.17). This was also true when host genders were analysed separately: genetic diversity pre- and post- treatment were not correlated in parasites from either females (p = 0.11) or males (p = 0.74).

**Fig 3:**
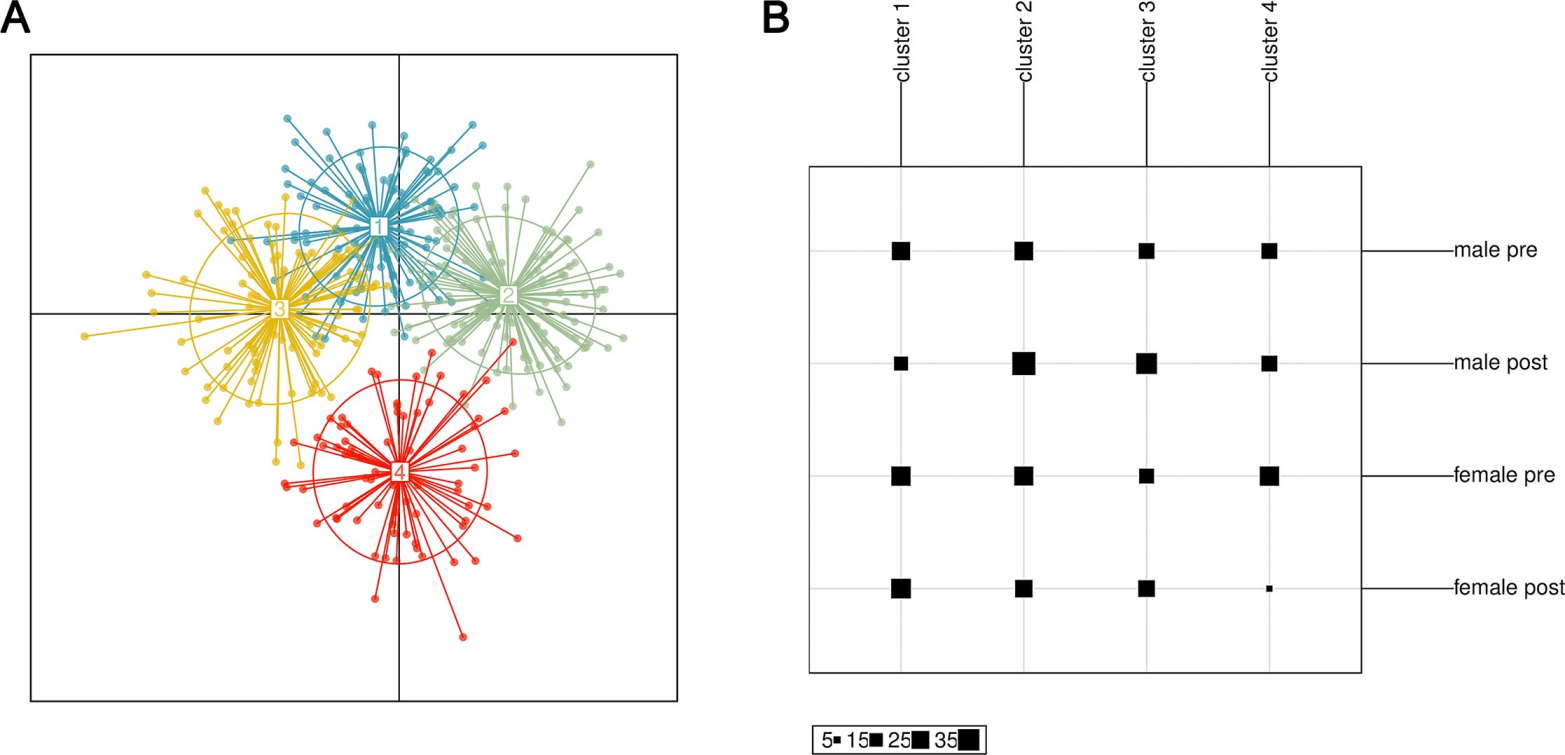
Population differentiation with discriminant analysis of principle components (DAPC). A. DAPC identified 4 clusters of parasite genotypes within the population sampled. B. However, these clusters were distributed relatively evenly between both genders and timepoints, suggesting no population structure determined by gender or treatment effects.

We asked whether population structure of schistosome parasites is differentiated by gender and how population changes with treatment. There was significant, yet low differentiation within the population—the mean Nei’s G_ST_ (fixation index for multi-locus data) between all individuals was 0.02 (p = 0.002). Despite this differentiation, there is not significant differentiation between genders or timepoints. Four distinct sub-population clusters were identified using discriminant analysis of principle components from the entire group of miracidia (Fig 3A). However, these clusters were not significantly correlated with treatment or gender (Fig 3B). Although there was population differentiation in parasites, differentiation was not associated with host gender or timing relative to treatment.

## Discussion

This study uses mixed methods to elucidate the impact of gender on exposure, risk behaviour and reinfection with intestinal schistosomiasis in Mayuge District, Uganda. Both male and female school-aged children contacted water daily through a variety of activities. The high abundance of water contact observed of both genders was mirrored by no significant differences in reinfection rates or parasite genetic diversity. There were slight differences in some of the actual water contact behaviours, but these differences represented a small proportion of the total water contact for the children. The household survey however indicates that these differences might become more pronounced as the individuals age. These results highlight that mixed methods can provide complementary findings, that can provide a more holistic understanding of disease transmission risk and therefore improved understanding of how to mitigate this.

We observe high rates of schistosomiasis transmission in this community, despite over a decade of MDA. In this study, 96% of the SAC in the longitudinal cohort were reinfected within five months post-treatment and parasite genetic diversity was very high within hosts both pre- and post-treatment. High reinfection rates are likely driven by children frequently contacting water during domestic, personal care, recreational, religious or commercial activities in this community. A high frequency of daily water contact has also been reported in a recent study from Senegal [45]. We found that frequency and duration of exposure to lake water was very similar between boys and girls. The only difference observed was that girls wash plates and fetch water for domestic use at a greater frequency than boys, whilst boys were the only ones that were observed to swim. This is consistent with an activity survey in Côte d’Ivoire, which also found boys swam more than girls [46] and where girls spend more time at domestic tasks. However, in our study we observed fewer gender-specific occupational activities, whilst the activity survey found boys engaging more in fishing and watering cattle and girls spending more time at the market. Our lack of observed occupational activities may be due to our recruitment in schools and therefore focus on school attending SAC, rather than just SAC in general, which could include non-enrolled and/or non-school-attending children. Although girls and boys may differ in the type of some activities they carry out, these different activities still commonly involve frequent water contact, in addition to the shared water contact activities (e.g. bathing) likely resulting in a similar net risk between girls and boys. Ongoing research includes additional qualitative data collection using focus group discussions and in-depth interviews to gain further insight into socio-economic determinants and other drivers of health behaviour in SAC. Furthermore, studies assessing differential risks of the various water contact activities and duration of water contact could contribute towards more understanding of exposure and risk of infection.

The similarity of water contact behaviours between genders is reflected in our epidemiological data presented here, with no significant differences by gender between reinfection risk for SAC. These findings support other epidemiological studies [11, 13]. After a period of clearance, egg production is observed less than six weeks later in 37% (n = 20) of the longitudinal cohort. Praziquantel treatment only affects adult worms and worm maturity is reached approximately six weeks after cercarial penetration of the human host. Therefore juvenile worms at the time of treatment are mostly unaffected by praziquantel. This could explain the timings of our first observed egg excretion after treatment. An alternative explanation is that praziquantel treatment causes embryostasis (cessation of egg production) and that the reduction in egg production is not always, or fully associated with adult death [47]. Genetic evidence for tolerant/resistant worms has been found in schistosomes in this region from the start of MDA [19] but more in-depth genetic studies to quantify the contribution of surviving adult worms compared to new worm infections are needed to disentangle these dynamics. In this high-transmission setting, we believe the high force of infection is possibly swamping out any of the small gender differences in observed and reported behaviour in children.

Analysis of parasite genetic diversity and population structure support minimal differences of infection risk between genders in SAC. If the differences in behaviours observed (more domestic exposure for girls, more swimming by boys) were exposing children to different parasites, we would expect genetic diversity to vary between the genders or population differentiation between parasites in these groups. This lack of difference in genetic diversity between genders is supported by other schistosome genetic diversity studies [16–18]. We find high genetic diversity in both genders and this recovers quickly, within five months, after observed successful treatment. This suggests that children are accumulating diverse parasite infections from the environment faster than annual MDA can control. We also found low, but significant population structure. This is surprising at such a small geographic scale and may be an artefact of the relative small number of miracidia (n = 286) and the high diversity of these loci. However, this differentiation does not partition in time or by host gender, however, suggesting gene flow between both genders and exposure to similar parasite genotypes. The smaller sample size here may be leading to these insignificant differences. A study in the same region at the start of MDA, 15 years ago with only praziquantel-naïve children, showed that males had parasites with significantly higher genetic diversity (although this was very small differences and dependent on age). These seemingly inconsistent findings between gender and parasite diversity could represent the changing epidemiology and risk of schistosomiasis in the era of MDA.

Based on survey data, the gender divisions in collecting and washing clothes is exacerbated in adults. Adult women are mainly responsible for fetching water and washing clothes, which was similar across various demographic and socio-economic variables within this community. However, the survey was structured so that it did not cover all water contact activities, for example occupational risks that may be more associated with adult men, such as fishing. Women contacting water mainly for domestic tasks with men contacting water for occupational tasks is supported by other studies in Uganda [6]. Although gender divisions are more pronounced in adults compared to SAC, we predict schistosomiasis infection is not significantly different between genders. Although not the same group in the household survey, a cross-sectional *S*. *mansoni* epidemiological survey in the same community found no significant impact of gender on risk of infection in univariate or multivariate analyses, even when age group (pre-SAC, SAC, and adults) was taken into account [48]. However, the self-reported duration of water contact and longer residence time in the village did significantly impact risk, further emphasising the importance of water contact and the high local risk for transmission. Future research on rates of reinfection in men and women could provide further insights in gender differences in risk among adults.

Although we used several complementary methods were used to evaluate behavioural and epidemiological risk to schistosomiasis, the study is still limited in its scope. Because of the investment in employing these methods, they were not repeated in other villages to test their robustness. However, Bugoto community is diverse and has been shown to be representative of other villages in the region [25]. Another limitation of this work is that the ethnographic, epidemiological and genetic studies were carried out on relatively small and non-overlapping groups of children. Participants were chosen for each sub-study based on specific needs and the inability to directly compare them can limit the conclusions drawn. However, many of the methods have been deployed in other settings and come to similar conclusions, so we do not expect these to be restrictive. Due to security, time and logistics constraints we were not able to observe early morning activities in SAC. We did include these through recall, and as the recall period was short we trust these data to be reliable and non-biased across individuals. However, we do not have level of submersion or duration data for these activities, and a few may be missed, which could lead to underestimation of the exposure. Finally, although the presence of the researcher could have altered observed behaviour, we aimed to limit this by building rapport, becoming a common site in and around the village whilst remaining as inobtrusive as possible, and working alongside a translator from the community who identified atypical behaviour.

Despite implementation of MDA in schistosomiasis-endemic areas, hotspots of high prevalence and rapid reinfection remain [49]. Improvements in treatment coverage through strengthening MDA programmes may help control some persistent transmission sites [50]. However it is increasingly recognised that additional, potentially more focused, interventions will be necessary to interrupt transmission and achieve longer lasting impacts on schistosomiasis endemicity [51]. A recently published cluster-randomised trial showed snail control and ‘classic’ WASH behaviour change interventions did not significantly boost the effect of MDA [52]. These intervention programs may need to be in place longer to contribute to reducing the disease burden. In addition, novel options could be explored such as understanding community members’ willingness to work and/or pay for different risk-mitigating community interventions. This could help devise the most effective, affordable and popular interventions and therefore most sustainable combination of interventions which might have a higher chance of being taken up, maintained and actually breaking transmission cycles [28].

Although both genders have high risk for schistosomiasis, results of the ethnographic and survey research highlight some behaviours that could be gender-specific targets for health promotion campaigns. The strong gender differences in water contact identified among adults were less strong among children, although girls were observed to fetch water more frequently and boys to swim more frequently. These different types of water contact may not produce substantive differences in volume of water contact, explaining the insignificance of gender as a determinant of parasitic load and diversity, however if total water contact is to be reduced, these could be targeted by gender group. In addition to previously found gender differences in areas such as drug treatment coverage [53], knowledge of, and attitudes towards, schistosomiasis [54] and host immunological and pharmacokinetic factors [55], disease control programmes should consider these high reinfection rates and the various purposes and settings in which children and adults are exposed to water, and be aware that some activities may be gender specific and may change over the life course.

## Supporting information

S1 Table Microsatellite markers used for population genetics. (DOCX)

S2 Table Number of miracidia sequenced from children pre- and post-treatment. (DOCX)

S1 Fig Timeline of epidemiology cohort (n = 55) sampling events. The entire SCHISTO_PERSIST cohort (n = 274) was also sampled at three timepoints (denoted in gold) in addition to the sampling points for the longitudinal cohort. The dashed red line indicates the initial praziquantel treatment.
(TIF)
