# Supplementary material for: Impacts of host gender on Schistosoma mansoni risk in rural Uganda—A mixed-methods approach
Source: PLoS Negl Trop Dis. 2020 May 13;14(5):e0008266. doi: 10.1371/journal.pntd.0008266 (PMC7219705; doi:10.1371/journal.pntd.0008266)
Supplement: S1 Table — (DOCX) [file pntd.0008266.s001.docx]

**S1 Table. Microsatellite markers used for population genetics.**

| **Panel 1** | **Repeat** | **Forward** | **Reverse** | **Reference** | **Dye** |
| --- | --- | --- | --- | --- | --- |
| SMD25 | CA | GATTCCCAAGATTAATGCC | GCCATTAGATAATGTACGTG | Durand et al. (2000) | 6-FAM |
| CA11-1 | GA-GT | TTCAAAACCATGAGCAATAGATAC | AGATGTTAGAAAGTGGTG | Blair et al. (2001) | NED |
| Ch5-A | AGA | CATTCATCGTAGAACATTGG | ATTATGGCTTCTTCATTTTCA | Criscione et al. (2009) | PET |
| R95529 | CAT | GTGATTGGGGTGATAAAG | CATGTTTCTTCAGTGTCC | Durand et al. (2000) | PET |
| Chr4E | TAT | AACGGTCCATTTTATTCTCC | AAATTGTTCCTAGCGTGTTC | Criscione et al. (2009) | VIC |
| L46951 | GAA | CAAACATATACATTGAATACAG | TGAATTGATGAATGATTGAAG | Durand et al. 2000 | VIC |
| SMD011 | GATA | TGTTTAAGTCGTCGGTGCTG | ACCCTGCCAGTTTAGCGTAG | Curtis et al. (2001) | VIC |
| **Panel 2** | **Repeat** | **Forward** | **Reverse** | **Reference** | **Dye** |
| SMD43 | GATA | CCCACCACAATTTATTGATCTC | GGGTCCTCCATTCCACTG | Curtis et al. (2001) | 6-FAM |
| Chr4G | AAT | TTGAGAACAAATGCTGCTAC | GATACTGCTTAAAATCGTCCTC | Criscione et al. (2009) | 6-FAM |
| SMC1 | AAT | TGACGAGGTTGACCATAATTCTAC | AACACAGATAAGAGCGTCATGG | Curtis et al. (2001) | 6-FAM |
| smCh1E | TAA | CTGGATCCAATCTTTTGAAG | CTTTTCCGGAATTCCATT | Criscione et al. (2009) | NED |
| smCH3H | ACT | GCACAGGCAAACAGTATCTC | TCTTTATTTACCATGCTGTACG | Criscione et al. (2009) | NED |
| Chr2A | ATC | CTCCTCTCGCTTTCTCTATTC | CAGGTTTCTCTAGCCAATTC | Criscione et al. (2009) | PET |
| Chr4H | AAT | GCTTTAACACACTGACTTTGTC | GAGGGTAATGAATCACTTGG | Criscione et al. (2009) | PET/NED |
| SMDA28 | GATA | CATGATCTTAGCTCAGAGAGCC | AGCCAGTATAGCGTTGATCATC | Curtis et al. (2001) | VIC |
| SMD28 | CAA | CATCACCATCAATCACTC | TATTCACAGTAGTAGGCG | Durand et al. (2000) | VIC |
| Chr3G | TA | GGTCATGTGGAATCTCAATC | TTAATGAAGTGACGATATAGCAG | Criscione et al. (2009) | VIC |
