# Supplementary material for: Impacts of host gender on Schistosoma mansoni risk in rural Uganda—A mixed-methods approach
Source: PLoS Negl Trop Dis. 2020 May 13;14(5):e0008266. doi: 10.1371/journal.pntd.0008266 (PMC7219705; doi:10.1371/journal.pntd.0008266)
Supplement: S2 Table — (DOCX) [file pntd.0008266.s002.docx]

**S2 Table. Number of miracidia sequenced from children pre- and post-treatment**

| **unique child id** | **age** | **gender** | **no. miracidia pre- treatment (10/17)** | **no. miracidia post- treatment (03/18)** |
| --- | --- | --- | --- | --- |
| BUG170607 | 6 | female | 15 | 10 |
| BUG170620 | 6 | male | 12 | 14 |
| BUG170625 | 6 | male | 12 | 10 |
| BUG170701 | 7 | female | 9 | 10 |
| BUG170714 | 7 | female | 11 | 8 |
| BUG170717 | 7 | male | 13 | 15 |
| BUG170901 | 9 | female | 14 | 14 |
| BUG170911 | 9 | female | 11 | 7 |
| BUG170921 | 9 | male | 15 | 12 |
| BUG171014 | 10 | female | 11 | 17 |
| BUG171017 | 10 | male | 11 | 14 |
| BUG171024 | 10 | male | 8 | 16 |
| BUG171312 | 13 | female | 13 | 13 |
| BUG171331 | 13 | female | 13 | 14 |
| BUG171328 | 13 | male | 10 | 13 |
| BUG171429 | 14 | male | 11 | 10 |
