# Supplementary figures and images for: Impacts of host gender on Schistosoma mansoni risk in rural Uganda—A mixed-methods approach
Source: PLoS Negl Trop Dis. 2020 May 13;14(5):e0008266. doi: 10.1371/journal.pntd.0008266 (PMC7219705; doi:10.1371/journal.pntd.0008266)

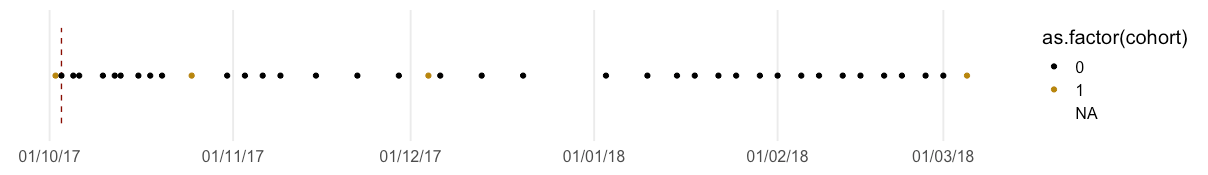

Supplement: S1 Fig — The entire SCHISTO_PERSIST cohort (n = 274) was also sampled at three timepoints (denoted in gold) in addition to the sampling points for the longitudinal cohort. The dashed red line indicates the initial praziquantel treatment. (TIF) [file pntd.0008266.s003.tif]
